# The neurocognitive processing mechanism of English subject-verb agreement by Chinese-speaking learners

**DOI:** 10.3389/fpsyg.2024.1402355

**Published:** 2024-07-04

**Authors:** Mingjun Wu, Miaomiao Li, Di Wu

**Affiliations:** School of Foreign Languages, Jiangsu University of Science and Technology, Zhenjiang, China

**Keywords:** subject–verb agreement, the thematic verb, omission errors, Chinese-speaking learners, the ERP technique

## Abstract

Determiner phrases (DPs), an overarching term, can be classified into two determiner types: referential determiner phrases (RDPs, e.g., the boy) and quantificational determiner phrases (QDPs, e.g., each boy). Using the event-related potential (ERP) technique, this study explored the modulation of RDP vs. QDP in the online processing of English subject–verb agreement with omission errors by Chinese learners of English, addressing the question of whether singular quantification increases or decreases Chinese learners’ sensitivity to agreement violations. The experiment manipulated the determiner type, specifically RDP vs. QDP, and grammaticality (grammatical vs. ungrammatical). The results indicated that similar to previous studies, a P600 effect was elicited in response to subject–verb agreement violations with omission errors, demonstrating that Chinese L2 learners are sensitive to such agreement violations. Additionally, the ERP patterns exhibited variations due to D-linking and number specification of RDP and QDP. Regarding D-linking, subject–verb agreement violations in the QDP conditions, necessitating integration of discourse-related knowledge, elicited laterally and frontally distributed P600 effects associated with integration complexity at the discourse level; however, non-D-linked referential determiners elicited the posteriorly-distributed P600 effects. Differences in number specification resulted in the distinctive P600 latencies and whether P600 was preceded by N400 or not. While both the RDP and QDP conditions exhibited the P600 effects, the onset latency of this effect in the number-unspecified RDP condition was 300 ms later compared to the number-specified QDP condition. Furthermore, an additional N400 component observed in the RDP condition suggests that L2 learners acquire morphologically complex subject–verb agreements by rote, treating them as unanalyzed chunks. This N400 component was absent in the QDP condition. From these results, the conclusion can be drawn that L2 learners are sensitive to the subject–verb agreement violations with omission errors, and L2 processing patterns of subject–verb agreement vary with different features of determiners, providing further evidence for the cue-based retrieval model during comprehension of grammatical sentences. Pedagogical implications are provided, and the future research direction is suggested.

## 1 Introduction

Successful language understanding demands establishing dependency between different linguistic constituents, even though they are often separated by intervening words, phrases, and sentences. The mental processes involved in encoding and retrieving linguistic information while establishing dependency is an active question in psycholinguistics. In order for language comprehension to be successful, the appropriate representations must be accessed from memory. Previous studies in linguistics and psycholinguistics emphasize that memory plays a significant role in the real-time processing of dependency (Miller and Chomsky, 1963). Over the last two decades, theories regarding the computational infrastructure of memory in language processing have become more sophisticated, particularly with regard to recognizing linguistic representations. One such framework is the cue-based retrieval model, which extends the computational principles of human recognition memory to language processing, providing an account for the establishment of syntactic dependency such as subject–verb agreement (Lewis et al., 2006; Wagers et al., 2009; Jäger et al., 2020). According to this model, subject–verb agreement, which is a ubiquitous phenomenon in some three-quarters of all the languages (Mallinson and Blake, 1981) and the most widespread type of agreement (Vigliocco et al., 1995), is established in real-time by retrieving subjects in working memory with features of verbs acting as retrieval cues. In English, subject–verb agreement is instantiated as a morphosyntactic number dependency between the subject and the verb. The subject responsible for controlling agreement features of the verb is termed the agreement controller, while the verb itself is denoted as the controlee (Bock and Miller, 1991; Wagers et al., 2009). For example, in (1), the number feature of the controlee *drive* is required to agree with that of the agreement controller. However, this requirement fails due to the number of disagreements between the singular agreement controller *car* and the plural controlee *drive*, resulting in subject–verb agreement violations.

(1) * The car drive fast on the race track.

Due to the fact that the morphological system used in Mandarin Chinese is severely simplified (Armstrong et al., 2018), the subject–verb agreement, despite a simple syntactic rule, seems to cause great difficulties for Chinese learners of English in both language production and comprehension, even for advanced learners and those living in English-speaking countries (Jiang, 2004, 2007; Lardiere, 2007). Recent studies have provided evidence that plural number specification of determiners (e.g., quantifiers and demonstratives) facilitates the computation of subject–verb agreement during online sentence comprehension, with greater sensitivity to violations specified by plural quantificational determiners (e.g., *many* and *some*) than those with referential determiners (e.g., *the*) (Tanner and Bulkes, 2015; Cheng et al., 2022). However, it is proposed that L2 learners commit more omission errors than commission errors (Lardiere, 2007); specifically, L2 learners tend to drop agreement markers, e.g., *look* instead of *looks*, more than they supply unnecessary agreement markers, e.g., *looks* instead of *look*. However, scarce research to date has explored how singular quantification (e.g., *each* and *every*) modulates the neural responses to violations of subject–verb agreement during online processing of omission errors. The current study, therefore, sought to bridge this gap by investigating the modulation of different determiners on the processing of English subject–verb agreement with omission errors by Chinese-speaking learners.

### 1.1 Different features of determiners

Determiners such as “the,” “this,” and “those” in the specifiers of noun phrases (NPs) violate the X-bar theory’s principle that all non-head material must be phrasal. To address this, Abney (1987) proposed the determiner phrases (DPs) hypothesis, asserting that DPs, traditionally named the NP, are phrases headed with the determiner. DPs, exemplified by “the boy” and “each boy,” can be categorized into referential determiner phrases (RDPs) and quantificational determiner phrases (QDPs). This categorization is based on the two intrinsic features possessed by determiners: number specification and D-linking (Cinque, 1990). In terms of number specification, plural quantifiers possess a plural number specification, and they can only merge with a plural noun (e.g., *many students*), not a singular noun (e.g., *many student). In contrast, the un-quantificational and number-ambiguous determiner (e.g., *the*) does not specify the grammatical number, and it co-occurs with either a singular noun or a plural noun. In this instance, the existence of a plural quantifier facilitates the processing of subject–verb agreement. Therefore, there is an increased sensitivity to subject–verb agreement violations, as evidenced by a larger P600 amplitude when the subject is explicitly marked as plural by a quantifier compared to when the subject remains unmarked by the determiner *the* (Tanner and Bulkes, 2015). D-linking, the other characteristic of determiners, distinguishes referential determiners *the* from quantifiers *each* (Pesetsky, 1987). The non-D-linked referential determiner *the* do not require introducing presupposed discourse representations, whereas D-linked quantifiers (e.g., *each* and *every*) require linking a set of presupposed entities (*boy* in *each boy*) to what quantifiers refer. Therefore, the interpretation of RDP *the boy* is purely syntactic, while the interpretation of QDP *each boy* imposes an additional requirement: integrating the syntactic and D-linked representations. This is parallel to the D-linked hypothesis (Avrutin, 2000; Sheppard et al., 2015), which accounts for the interpretation of different types of *wh*-questions, specifically, *what*-questions and *which*-questions. Therefore, we hypothesize that comprehenders’ processing of subject–verb agreement may be influenced by the D-linking feature of DPs.

### 1.2 The cue-based retrieval model accounting for subject–verb agreement

**Figure 1:**
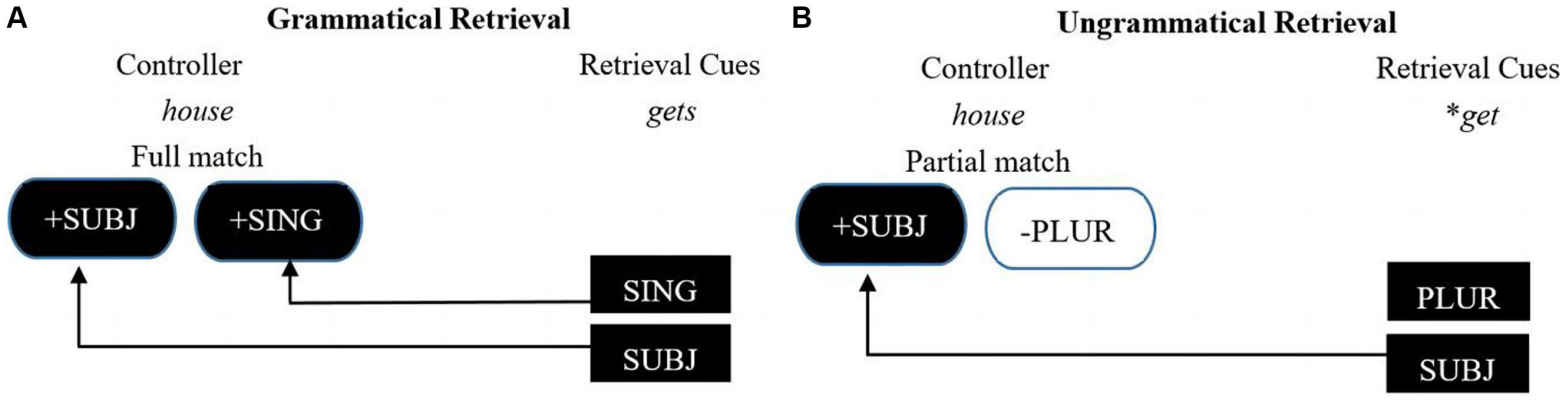
The cue-based retrieval model. Figures **(A)** and **(B)** illustrate how cue-based parsing works to establish the dependency between a verb (“gets/*get”) and a subject (“house”).

One well-supported model of parsing of dependency is the cue-based retrieval model (Lewis et al., 2006; Wagers et al., 2009; Jäger et al., 2020). According to the model, the features of the controller are stored in memory, and then the features of the controllee serve as retrieval cues to search for the controller in working memory. When a feature mismatch is detected, a reanalysis process is initiated to fix the mismatch problem. Regarding subject–verb agreement, the number feature, like [+Singular], and the structural feature, like [+Nominative] or [+ Subject], are the two types of retrieval cues provided by the verb (Wagers et al., 2009), as shown in (2) and (3). According to the cue-based retrieval model (see Figure 1), after the singular subject noun, *the house,* is encountered, the number feature [+Singular] and structural feature [+Nominative] or [+Subject] are stored in memory. If the verb is singular, as in (2), it is congruent with the subject *the house* in both number and structural features. In this case, agreement dependency between the subject and the verb is successfully constructed. However, if the verb is plural, as in (3), it does not agree with the number feature [+Singular] of the subject. As a result, parsers must initiate a reanalysis process to retrieve a matching subject in working memory. Subject–verb agreement violations elicit a P600 effect as an index of processing difficulty in subject–verb dependency processing (Kaan, 2002; Kaan and Swaab, 2003).

(2) The house gets repainted every summer.

(3) *The house get repainted every summer.

### 1.3 Empirical studies on L1 English subject–verb agreement

For decades, extensive research has been conducted on English native speakers’ acquisition and processing of subject–verb agreement using various methods (Bock and Miller, 1991; Osterhout and Mobley, 1995; Wagers et al., 2009; Dillon et al., 2013; Tanner and Bulkes, 2015; Dube et al., 2016). The results of eye-tracking tasks and moving-window reading experiments suggest that native speakers typically exhibit more regressive eye movements or longer reading times for sentences that contain subject–verb agreement violations in comparison to grammatical sentences (Wagers et al., 2009; Dillon et al., 2013). In addition, much neurocognitive research using ERPs has shown that subject–verb agreement violations elicit a P600 effect, occurring approximately 500 ms after the onset of the stimulus, which reflects a late stage of reanalysis, which operates on qualitatively distinct information sources (Kuperberg, 2007; Bornkessel-Schlesewsky and Schlesewsky, 2008). Crucially, the P600 exhibits high sensitivity to subtle processing distinctions, as evidenced by variations in the scalp topography, onset, and amplitude of P600 effects (Kaan and Swaab, 2003; Nevins et al., 2007; Gouvea et al., 2010; Tanner and Van Hell, 2014; Tanner and Bulkes, 2015). For example, distinctions exist between “early P600” and “late P600” (Hagoort et al., 1999), as well as between “frontal P600” and “parietal P600” (Friederici, 2002; Kaan and Swaab, 2003). The onset latency of P600 is associated with L2 proficiency in studies of L2 processing (Hahne et al., 2006; Rossi et al., 2006), reflecting the difficulty of detecting a mismatch and completing the reanalysis process (Friederici, 1998). Scalp distribution differences of P600 reflect distinctive underlying cognitive processes, with frontally distributed P600 correlating with an increase in discourse complexity (Kaan and Swaab, 2003) and posteriorly distributed P600 correlating with syntactic processing difficulties (Tanner et al., 2017). Another ERP component is the N400, which occurs at approximately 300–500 ms after stimulus onset, generally reflecting the processing of novel word combinations or word sequence probabilities (Kutas and Hillyard, 1984; Osterhout et al., 2006). However, in some studies, both L1 and L2 speakers exhibit the N400 for morphosyntactic contexts (McLaughlin et al., 2010; Tanner et al., 2013; Bian et al., 2021).

There is only one L1 study examining how the determiner type modulates the processing of English subject–verb agreement (Tanner and Bulkes, 2015). Tanner and Bulkes (2015) used ERPs to investigate how plural number-specified determiners (e.g., *many* and *some*) of the subject influence subject–verb agreement comprehension by native English speakers (e.g., The/Many teachers meet/*meets with the lawyer at the pub). Concerning commission errors, they find that subject–verb agreement violations, both in the RDP and QDP conditions, elicited the P600 effect. However, P600 effects were larger in the QDP than in the RDP condition, indicating heightened sensitivity to the number agreement violations.

Findings from these L1 studies indicate that native speakers are consistently sensitive to agreement violations, as demonstrated by longer reading times or more regressive eye movements in behavioral studies and the P600 effect in neurolinguistic research on subject–verb agreement violations in comparison to grammatical sentences.

### 1.4 Empirical studies on L2 English subject–verb agreement

A large amount of literature has examined subject–verb agreement processing by L2 learners (Jiang, 2004; Chen et al., 2007; Tanner et al., 2012; Armstrong et al., 2018; Bian et al., 2021). Attempts have been made to systematically investigate such factors as number attraction, L1-L2 similarity, and proficiency to establish whether or how they may impact the processing of subject–verb agreement during online sentence comprehension (Tokowicz and MacWhinney, 2005; Tanner et al., 2012, 2013). Tanner et al. (2012) employed ERPs to investigate the influence of attraction interference on the processing of subject–verb agreement in both late proficient Spanish–English bilinguals and monolingual English speakers by manipulating the attractor noun’s number which occurred between the subject noun and the verb (e.g., The writer of the script/scripts was/*were very popular). The results suggested that both native English speakers and Spanish–English bilinguals showed a P600 effect in response to subject–verb agreement violations, but the overall size of the P600 was smaller, indicating an attraction effect. Tokowicz and MacWhinney (2005) found that while bilinguals exhibited P600 patterns in response to violations for constructions similar in the L1 and the L2, no P600 effects were elicited for constructions that differed in the L1 and the L2. Tanner et al. (2013) examined how language proficiency influences the processing of German subject–verb agreement in English-speaking learners of L2 German using ERPs. They observed the presence of systematic individual variations in the L2 learners’ neural responses to number agreement violations. While highly proficient L2 learners show the P600 effect, low-proficiency L2 learners display the N400–P600 biphasic effect in response to the same set of agreement violations.

Since Chinese does not manifest the agreement relationship between distinct constituents within a sentence (e.g., subject–verb agreement), even advanced Chinese L2 learners, who are regarded as highly proficient and effective users of the language, encounter challenges in the comprehension and production of English subject–verb agreement (Lardiere, 1998; Jiang, 2004; Armstrong et al., 2018). In production, Chinese learners’ difficulty with English number agreement is evidenced in a longitudinal study that collected a highly proficient L1 Chinese speaker’s naturalistic production data over a period of 8.5 years (Lardiere, 1998). The naturalistic production data demonstrated that in obligatory contexts, the third singular -s morpheme was produced by the participants in merely 4.54% of instances with non-past thematic main verbs. Additionally, in comprehension, ample findings provide evidence for Chinese learners’ persistent difficulties in processing English agreement (Chen et al., 2007; Armstrong et al., 2018; Bian et al., 2021).

While most research investigates subject–verb agreement with the copular verb *be*, only one ERP study examines how quantificational cues to the subject DP modulate Chinese learners’ sensitivity to the subject–thematic verb agreement violation processing (Armstrong et al., 2018). Armstrong et al. (2018) used ERPs to examine how plural quantification of the subject DP interacts with English subject–verb agreement (e.g., The/Many tropical birds sing/*sings in the exotic pet store.). They find that similar to native speakers (Tanner and Bulkes, 2015), Chinese learners exhibit a robust P600 effect in response to agreement violations relative to subject–verb agreement sentences. However, the amplitude of the P600 effect elicited by agreement violations differs between Chinese L2 learners and native English speakers (Tanner and Bulkes, 2015). Specifically, when comprehending the sentences involving subject–verb agreement violations, the L2 learners exhibit a larger P600 amplitude for the unquantified subject DP, whereas native English speakers show a larger amplitude for the quantified subject DP.

### 1.5 The current study

It is necessary to note that the only two studies (Tanner and Bulkes, 2015; Armstrong et al., 2018), which focus on the modulation of the plural quantification to subject–verb agreement processing, have weak pedagogical implications due to the fact that L2 learners seldom commit commission mistakes (Lardiere, 2007). To be specific, when the subject is singular, L2 learners are prone to omit the third person singular −*s* inflection, whereas when the subject is plural, L2 learners are less likely to mistakenly add redundant -*s* marking on the verb. In addition, in studies of the subject–verb agreement using the number attraction paradigm, the predicate is often the copular verb “be” (Tanner et al., 2012; Bian et al., 2021), which does not address the “omission errors” with thematic verbs that Chinese L2 learners commonly make. This lack of representativeness also limits the pedagogical implications. Therefore, the current study is motivated to extend the existing research using plural quantificational subject to singular quantificational subject and aims to examine its role in processing omission errors of subject–verb agreement by recording L2 comprehenders’ ERP responses to grammaticality crossed with two determiner types. To be specific, we aim to address the following two research questions.

(1) Are Chinese learners sensitive to the subject–verb agreement violations with omission errors? If so, do L2 learners exhibit P600 effects similar to native speakers?

Prediction 1: We hypothesize that L2 learners exhibit the native-like P600 effects.

(2) Does singular quantification increase or decrease Chinese learners’ sensitivity to the subject–verb agreement violations? If so, how does singular quantification affect the onset latency or distribution of the P600 effect?

Prediction 2: We hypothesize that singular quantification increases Chinese learners’ sensitivity to English subject–verb agreement as evidenced by the earlier P600 onset latency in the QDP condition compared to RDP. In addition, we predict that the processing of subject–verb agreement may be influenced by the D-linking feature of DPs, as evidenced by the frontal distribution of the P600 effect.

## 2 Materials and methods

### 2.1 Participants

The experiments were conducted in accordance with the Declaration of Helsinki, and all procedures were carried out with the written informed consent of the participants. The research has been approved by an *ad hoc* human ethics review committee from the School of Foreign Languages, Jiangsu University of Science and Technology. Before running our experiments, *a priori* power analysis was conducted using G*Power for sample size estimation (Faul et al., 2007). Based on the effect size of f^2^ = 0.25, the α level of 0.05, and the experimental design of the experiment, the G*Power calculation revealed that 36 participants could achieve a sufficient statistical power of 0.95. We recruited 36 undergraduate students majoring in English who learned English as a second language from a university in China (mean age 20 years, range 18–23) with no immersion experience. All of the participants got course credit for taking part, provided their written informed consent, and completed a language history questionnaire. They started to learn English at approximately 9 years old and had learned English for over 12 years when the experiment was conducted. They had normal or corrected-to-normal vision and were strongly right-handed (Oldfield, 1971); none of them reported a history of neurological disorder, head injuries, or taking psychoactive medication. Approximately half of them passed the Test for English Majors Band 4 (TEM-4), which is administered yearly to intermediate English majors by the official National Advisory Commission on Foreign Language Teaching in Higher Education in China and is widely acknowledged across the country as evidence of English proficiency. After the experiment, all participants were administered a proficiency test of the Grammar Part in the Oxford Placement Test (OPT) (Allan, 2004). Their raw scores varied from 54 to 84 (Mean = 71.75, SD = 6.78), corresponding to the proficiency level of A2 to C1 of the Common European Framework of Reference (CEFR), which is a widely adopted guideline used to describe the language proficiency levels of learners of foreign languages across Europe and increasingly in other countries. Relative to native speakers (89%) (Tanner and Bulkes, 2015) and L2 learners with intermediate to advanced proficiency (77%) (Armstrong et al., 2018), the acceptability judgment accuracy (72%) of the present study was lower. Based on the sentence judgment accuracy, OPT scores, and the pass rate of TEM-4, our participants’ English proficiency is judged to be intermediate.

### 2.2 Materials

The 120 sentences for the experiment were adapted from those used by Tanner and Bulkes (2015) and Armstrong et al. (2018). We modified the materials by changing all plural subjects to singular and replacing plural quantifiers such as “many/some” with singular ones like “each/every.” This adaptation does not affect the reliability of the sentences. Determiner Type (RDP vs. QDP) and Grammaticality (grammatical vs. ungrammatical) were crossed in a 2 × 2 design (see Table 1). The sentences included a singular DP subject, which was singularly marked by a quantifier (*each* or *every*), or included an unquantified referential determiner, *the,* ambiguous for number, and the verb either agreed or disagreed with the subject. The thematic verbs never occurred at the end of the sentence. The sentences were distributed across four separate lists through a Latin square design so that no participant was presented with two conditions of the same sentence set.

**Table 1:** Example stimuli and Chinese translations in each experimental condition.

Determiner type	Example sentence
RDP	The TV writer needs to write a new script each week. 这位电视编剧**需要**每周写一个新剧本。
	*The TV writer need to write a new script each week. 这位电视编剧**需要**每周写一个新剧本。
QDP	Every TV writer needs to write a new script each week. 每位电视编剧**需要**每周写一个新剧本。
	*Every TV writer need to write a new script each week. 每位电视编剧**需要**每周写一个新剧本。

RDP, the referential determiner phrase; QDP, the quantificational determiner phrase.

### 2.3 Procedure

Each participant was randomly assigned to a stimulus list and tested individually in the experiment, lasting approximately 2.5 h. They were told to relax and minimize their body movements and blinks when reading these sentences. Each sentence contained the following sequence of events. At first, a fixation cross was presented for an unlimited time. Once the participants were ready to begin reading, they started by pressing the keys on the keyboard. Then, the sentences were displayed on the screen word by word, with each word presented onscreen for 500 ms, followed by a 350 ms interstimulus interval. Sentences were followed by a blank screen presented for 950 ms. Finally, a prompt appeared on the screen asking participants to judge the acceptability of the sentences by pressing the Y key for being grammatically and semantically acceptable, and the N key for being unacceptable. Participants were given six practice trials with three syntactically or semantically ill-formed sentences to acquaint themselves with the experiment. Participants were encouraged to relax or blink when the fixation cross was presented onscreen between each trial. The task was divided into eight blocks, and participants were given a short break between each block.

### 2.4 Data acquisition and analysis

Continuous EEG data were recorded using 32 scalp electrodes, digitized with a sampling rate of 500 Hz (Electro-cap International 10–20 system). It was filtered with online analog 0.016- to 100-Hz bandpass and offline with a 0.05-Hz low cutoff and 40-Hz low cutoff filter. Electrode impedances at the scalp were held below 5 KΩ during data collection. The offline processing was performed by BrainVision Analyzer (Brain Products, Munich, Germany), with the data re-referenced offline to the algebraic average of the activity measured in the left mastoid (TP9) and right mastoid (TP10). Epochs ranging from −200 ms (baseline) to 1,200 ms relative to critical word onset were created. A combination of automatic and manual rejection was employed to remove bad epochs containing artifacts such as EOG, EMG, drift, or technical problems. The software automatically identified artifacts based on the parameters. After the automatic identification, we performed a manual check to determine whether the artifacts were caused by muscle activity, eye movements, or drift, or if they were due to objective factors unrelated to our participants, such as equipment malfunction, strong electrical interference, or electrode issues. If an artifact was caused by a damaged electrode at a non-critical site, we performed electrode interpolation to address it. For instance, if an artifact was caused by a damaged electrode at a non-critical site, we performed electrode interpolation to address it. ERPs time-locked to the onset of the thematic verb were averaged with the 200 ms prestimulus baseline. Based on the visual inspection of grand averages and previous findings, mean amplitudes for analyses were done separately for time windows of 350–500 ms, 700–950 ms, and 1,000–1,100 ms. The 350–500 ms time window was selected for testing the N400 component, which is associated with difficulty in plausibility processing or semantic integration (Nieuwland, 2019). Additionally, previous studies demonstrate that both L1 and L2 speakers show the N400 effects for morphosyntactic contexts (McLaughlin et al., 2010; Tanner et al., 2013; Bian et al., 2021). The 700–950 ms time window was chosen for testing the P600 component, which reflects the reanalysis and syntactic processing difficulty of the whole sentence containing different sources of information (Kuperberg, 2007). The 1,000–1,100 ms time window was selected for testing the late P600 component, which is related to the reanalysis or repair of morphosyntactic violations.

Based on the scalp distribution of the language-related ERP components, specifically N400 and P600, reported in previous studies (Kutas and Hillyard, 1984; Friederici, 2002; Kaan and Swaab, 2003; Bian et al., 2021), we performed the repeated measures analyses of variance (ANOVA) for midline and lateral electrodes, respectively. For midline electrode analyses, ANOVA variables were Grammaticality, Determiner Type, and Electrodes (Fz vs. Cz vs. Pz). For lateral electrode analyses, four clusters of lateral electrodes (right frontal: F4, FC2, FC6; right posterior: CP2, CP6, P4; left frontal: F3, FC5, FC1; and left posterior: CP5, CP1, P3) were our regions of interest. Hemisphere (left and right) and Anteriority (anterior and posterior) were the ANOVA variables alongside Grammaticality and Determiner Type. We conducted the Greenhouse–Geisser correction whenever the assumption of sphericity was violated and reported corrected *p*-values. Only statistical effects involving the manipulated experimental factors (Grammaticality and Determiner type) and their interaction with topographic factors are reported.

## 3 Results

### 3.1 Behavioral data

The average grammaticality judgment accuracy and mean reaction time across four conditions were presented in Table 2. The accuracy (72%) indicated that participants had a solid grasp of the grammar in the target language and could identify agreement violations. The ANOVA for accuracy across the four conditions showed a significant difference (significant at alpha = 0.05 level): the main effect of Grammaticality (*F*(1,35) = 5.166, *p* = 0.029, *η_p_^2^* = 0.129) and Determiner Type (*F*(1, 35) = 7.324, *p* = 0.010, *η_p_^2^* = 0.173). The ANOVA for reaction time revealed a main effect of Grammaticality (*F*(1,35) = 7.623, *p* = 0.009, *η_p_^2^* = 0.179). The ANOVA results for both accuracy and reaction time showed no significant interaction effects.

**Table 2:** Average accuracy and standard deviation for grammaticality judgment by Chinese L2 learners.

Condition	Grammatical		Ungrammatical	
	Average accuracy (%)	Reaction time (ms)	Average accuracy (%)	Reaction time (ms)
RDP	77(3.1)	2,207 (1479)	63 (7.4)	1,669 (996)
QDP	77(4.0)	1948 (1318)	70 (7.9)	1707 (1206)

### 3.2 ERP data

**Figure 2:**
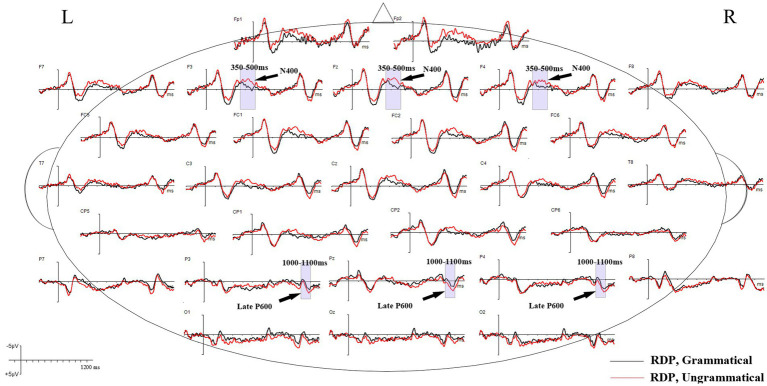
Grand mean ERP waveforms for grammatical (black lines) and ungrammatical (red lines) verbs in the RDP condition. The negative voltage is plotted up. The waveforms depict 200 ms of prestimulus and 1,200 ms of poststimulus activity; each tick mark represents 100 ms of time. The vertical calibration bar shows ±5 μV of activity.

**Figure 3:**
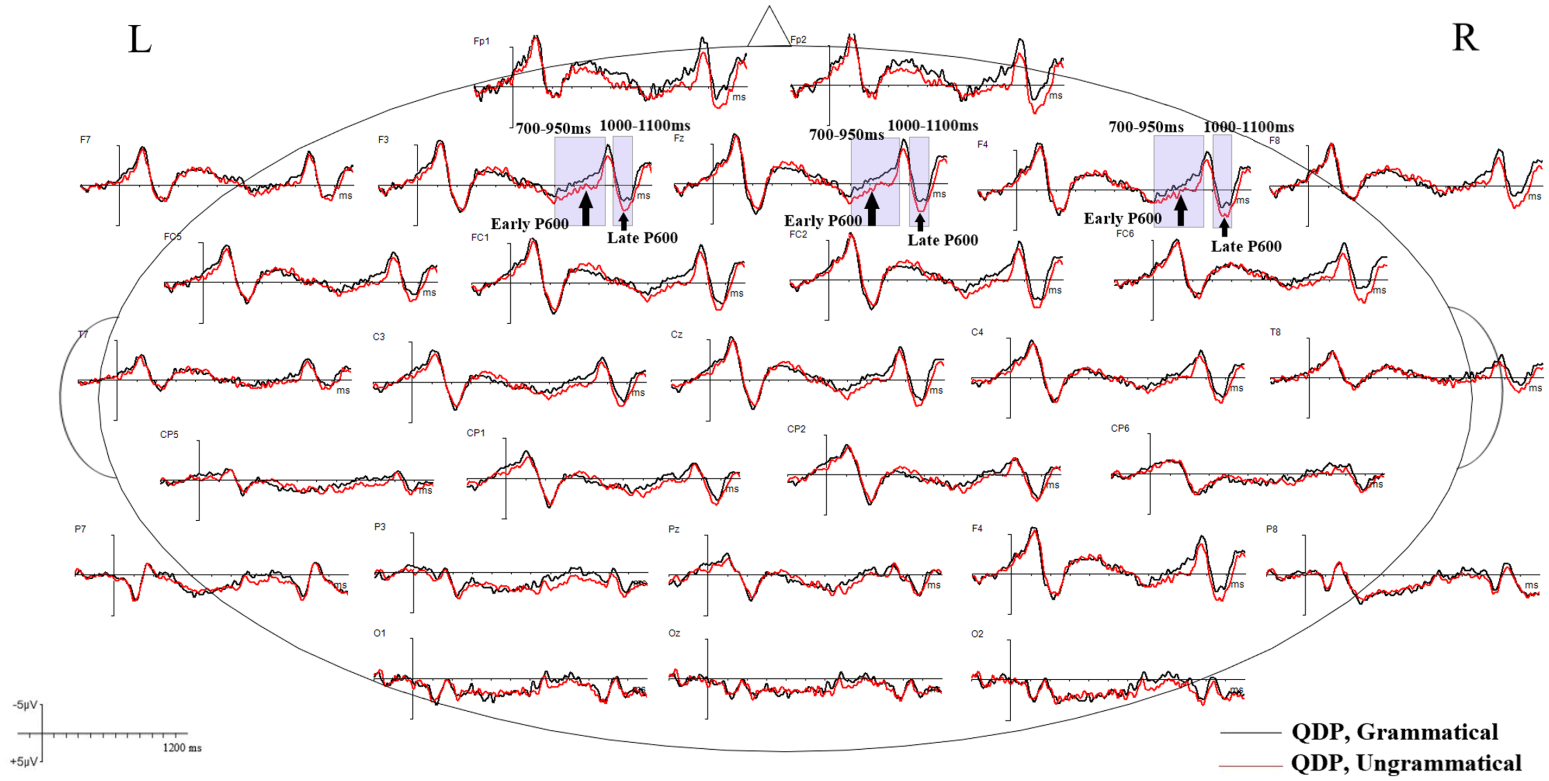
Grand mean ERP waveforms for grammatical (black lines) and ungrammatical (red lines) verbs in the QDP condition. The negative voltage is plotted up. The waveforms depict 200 ms of prestimulus and 1,200 ms of poststimulus activity; each tick mark represents 100 ms of time. The vertical calibration bar shows ±5 μV of activity.

Grand mean waveforms of the neural responses to grammatical and ungrammatical conditions are shown in Figures 2, 3 for the RDP and QDP conditions from 200 ms before the onset of the thematic verb up to 1,200 ms. As illustrated in Figure 2, the grand mean ERPs in the RDP condition suggest that agreement violations (e.g., *Every/The TV writer need to write a new script each week.) elicit a clear negative component (N400) during 350–500 ms, relative to grammatical sentences (e.g., Every/The TV writer needs to write a new script each week.). This component is absent in previous studies that focus on the plural quantification (Tanner and Bulkes, 2015; Armstrong et al., 2018); additionally, agreement violations in the RDP condition elicit a late positive component (late P600) during 1,000–1,100 ms, relative to grammatical sentences. In contrast, as illustrated in Figure 3, the ungrammatical condition in the QDP condition elicits an early positive wave (early P600) and a late positive wave (late P600), which are frontally distributed compared to the baseline grammatical sentences. This qualitatively similar P600 effect is consistent with previous studies (Tanner and Bulkes, 2015; Armstrong et al., 2018).

#### 3.2.1 Time window 350–500 ms (N400)

For midline electrodes, ANOVA results demonstrated a main effect of Grammaticality (*F*(1,35) = 7.288, *p* = 0.011, *η_p_^2^* = 0.172), showing that ungrammatical sentences elicited more negative ERP responses relative to grammatical sentences. A significant interaction of Electrodes with Determiner Type (*F*(2, 70) = 5.623, *p* = 0.010, *η_p_^2^* = 0.138) revealed that there was a significant difference between the RDP condition and the QDP condition at the Pz electrode. For lateral sites, ANOVA results indicated the main effect of Grammaticality (*F*(1,35) = 7.831, *p* = 0.008, *η_p_^2^* = 0.183), showing that the brain responses to ungrammatical sentences were more negative-going than to grammatical sentences.

#### 3.2.2 Time window 700–950 ms (Early P600)

For midline electrodes, ANOVA results revealed a main effect of Grammaticality (*F*(1,35) = 4.837, *p* = 0.035, *η_p_^2^* = 0.121) in which the mean amplitude of ungrammatical sentences was more positive than that of grammatical sentences. For lateral sites, the ANOVA results revealed a marginally significant main effect of Grammaticality (*F*(1,35) = 3.917, *p* = 0.056, *η_p_^2^* = 0.101). The marginally significant three-way interaction between Anteriority, Determiner Type, and Grammaticality (*F*(1,35) = 3.995, *p* = 0.053, *η_p_^2^* = 0.102) indicated that the QDP condition elicited a frontally distributed P600 (*p* = 0.036), whereas the RDP condition did not.

#### 3.2.3 Time window 1,000–1,100 ms (Late P600)

For midline electrodes, ANOVA results revealed a main effect of Grammaticality (*F*(1,35) = 8.932, *p* = 0.005, *η_p_^2^* = 0.203), showing that subject–verb agreement violations elicited a P600 effect. There was a marginal significance in the three-way interaction between Electrodes, Determiner Type, and Grammaticality (*F*(2, 70) = 3.038, *p* = 0.073, *η_p_^2^* = 0.080), indicating that the RDP condition at Pz showed a significant P600 effect (*p* = 0.048), whereas the QDP condition did not. For lateral sites, ANOVA results showed the main effect of Grammaticality (*F*(1,35) = 10.392, *p* = 0.003, *η_p_^2^* = 0.229). The three-way interaction between Anteriority, Determiner Type, and Grammaticality was significant (*F*(1,35) = 4.813, *p* = 0.035, *η_p_^2^* = 0.121), showing that the QDP condition elicited a frontally distributed P600 (*p* = 0.028), whereas the RDP condition elicited a posteriorly distributed P600 (*p* = 0.055).

**Figure 4:**
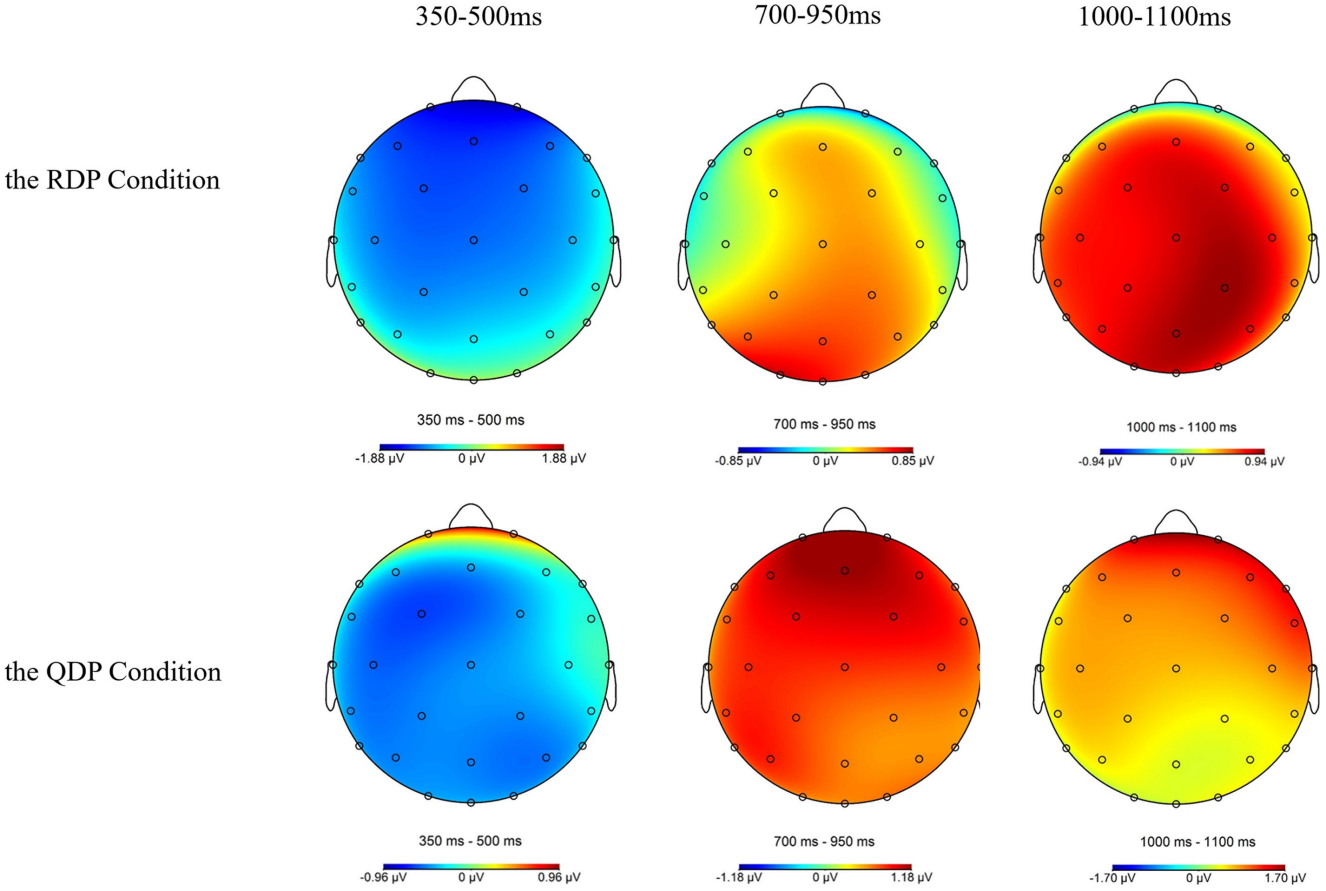
Scalp topographies for the difference between grammatical and ungrammatical (ungrammatical minus grammatical) words under the RDP condition (top panel) and the QDP condition (bottom panel) in 350–500 ms, 700–950 ms, and 1,000–1,100 ms time windows. RDP, referential determiner phrase; QDP, quantificational determiner phrase.

All of the statistical analyses for midline and lateral electrodes in the time windows of 350–500 ms, 700–950 ms, and 1,000–1,100 ms are summarized in Table 3, and simple effect results are summarized in Table 4 with significant and marginally significant results bolded. Scalp topographies for the difference wave displayed in Figure 4 clearly illustrated that P600 in RDP conditions distributed posteriorly while P600 in QDP conditions distributed laterally and frontally.

**Table 3:** Summary of statistical analyses.

	Time windows											
	350-500 ms				700-950 ms				1,000-1100 ms			
Midline	df	*F*	*η_p_^2^*	*p*	df	*F*	*η_p_^2^*	*p*	df	*F*	*η_p_^2^*	*p*
Grammaticality	(1,35)	7.29	0.17	**0.01**	(1,35)	4.84	0.12	**0.04**	(1,35)	8.93	0.20	**0.01**
Electrodes × Determiner Type	(2,70)	5.62	0.14	**0.01**	(2,70)	0.85	0.02	0.40	(2,70)	0.95	0.03	0.37
Electrodes × Determiner Type × Grammaticality	(2,70)	1.85	0.05	0.18	(2,70)	2.31	0.06	0.12	(2,70)	3.04	0.08	**0.07**
Lateral												
Grammaticality	(1,35)	7.83	0.18	**0.01**	(1,35)	3.92	0.10	**0.06**	(1,35)	10.39	0.23	**0.00**
Anteriority × Determiner Type	(1,35)	9.08	0.21	**0.01**	(1,35)	2.41	0.06	0.13	(1,35)	1.73	0.05	0.20
Anteriority × Determiner Type × Grammaticality	(1,35)	2.34	0.06	0.14	(1,35)	4.00	0.10	**0.05**	(1,35)	4.81	0.12	**0.04**

Significant at alpha = 0.05 level. Significant and marginally significant results are bolded.

**Table 4:** Results of simple effects.

Time Windows	Region	RDP: grammatical vs. ungrammatical								
		Left			Mid			Right		
		*t*	df	*P*	*t*	df	*p*	*t*	df	*p*
350–500 ms	F	2.747	35	**0.009**	2.756	35	**0.009**	2.177	35	**0.036**
	FC	2.405	35	**0.022**	2.508	35	**0.017**	2.199	35	**0.035**
	CP	1.905	35	**0.065**				1.641	35	0.110
	P	2.022	35	**0.051**	1.252	35	0.219	1.305	35	0.201
700–950 ms	F	−0.705	35	0.485	−1.400	35	0.170	−0.540	35	0.593
	FC	−0.042	35	0.966	−1.172	35	0.249	−0.016	35	0.987
	CP	−0.830	35	0.412				−1.032	35	0.309
	P	−0.892	35	0.379	−1.108	35	0.275	−1.180	35	0.246
1,000–1,100 ms	F	−1.855	35	**0.072**	−2.072	35	**0.046**	−1.190	35	0.242
	FC	−1.342	35	0.188	−2.084	35	**0.045**	−1.311	35	0.198
	CP	−1.854	35	**0.072**				−1.971	35	**0.057**
	P	−1.555	35	0.129	−1.875	35	**0.069**	−1.663	35	0.105
Time Windows	Region	QDP:grammatical vs. ungrammatical								
		Left			Mid			Right		
		*t*	df	*P*	*t*	df	*p*	*t*	df	*p*
350–500 ms	F	0.886	35	0.382	0.813	35	0.422	0.431	35	0.669
	FC	1.144	35	0.260	0.723	35	0.474	0.482	35	0.633
	CP	1.286	35	0.207				0.931	35	0.358
	P	0.990	35	0.329	0.953	35	0.347	1.061	35	0.296
700–950 ms	F	−1.625	35	0.113	−1.892	35	**0.067**	−1.858	35	**0.072**
	FC	−1.882	35	**0.068**	−1.138	35	0.263	−2.032	35	**0.050**
	CP	−1.671	35	0.104				−1.202	35	0.237
	P	−1.665	35	0.105	−0.680	35	. 501	−1.161	35	0.254
1,000–1,100 ms	F	−1.766	35	**0.086**	−1.556	35	0.129	−1.994	35	**0.054**
	FC	−1.803	35	**0.080**	−1.267	35	0.213	−2.218	35	**0.033**
	CP	−1.415	35	0.166				−1.081	35	0.287
	P	−1.400	35	0.170	−0.465	35	0.645	−0.940	35	0.354

Significant at alpha = 0.05 level. Significant and marginally significant results are bolded.

## 4 Discussion

The current study aims to examine whether singular quantification modulates the processing of English subject–verb agreement by L2 Chinese learners of English. Specifically, we examined (i) whether L2 learners can exhibit a P600 effect in response to the subject–verb agreement violations and (ii) whether the computation of subject–verb agreement violations with omission errors varies as a function of different determiner types. Two major findings in the present study are as follows. P600 is elicited in response to omission errors, which indicates that L2 learners are sensitive to subject–verb agreement violations. Moreover, different features of determiners modulate subject–verb agreement processing, resulting in different ERP patterns.

### 4.1 Chinese learners’ sensitivity to omission errors in L2 subject–verb agreement

Our first finding indicated that subject–verb agreement violations with omission errors elicited a P600 effect, showing that Chinese L2 learners are sensitive to agreement information during online comprehension. Previous ERP research on subject–verb agreement processing shows that a P600 effect is reliably elicited in native speakers (Osterhout and Mobley, 1995; Tanner et al., 2013; Tanner and Bulkes, 2015). Thus, the P600 effect is taken as a crucial ERP index for morphosyntactic processing in native speakers, especially for the processing of subject–verb agreement. It is proposed that the emergence of the P600 effect in L2 learners constitutes evidence of the attainment of nativelike grammatical processing (Tanner et al., 2013; Armstrong et al., 2018). In this aspect, Chinese learners have grammaticalized English subject–verb agreement rules and can incorporate these rules into their real-time language processing system.

It must be recognized that the previous accounts for the P600 component have ignored the important role of working memory in establishing dependencies, and it is worthwhile to take into account the potential integration of our results with the current P600 interpretation. We argue that P600 may be elicited when retrieval fails to establish subject–verb agreement dependency. Retrieval failure gives rise to an increase in memory loads when subject–verb agreement violations are encountered, which is in line with the cue-based retrieval account (Lewis et al., 2006; Wagers et al., 2009; Vasishth and Engelmann, 2022). According to this account, features of the verb are used as retrieval cues to search for the target in working memory. When the subject number matches the verb number, agreement dependency is successfully established; however, when mismatches occur, a reanalysis process is initiated to fix the mismatch problem. For example, in 4(a), the retrieval cues [+Singular] and [+Subject] provided by the verb *performs* agree with [+Singular] and [+Subject] features of the DP *the/every actor*, thus subject–verb agreement dependency is successfully formed. However, in 4(b), the retrieval cue [-Singular] provided by the verb *perform* disagrees with [+Singular] of the subject, and the parser detects a number disagreement between the subject and the verb, and, therefore, a reanalysis process is initiated, instantiating the P600 effect.

4 (a) The/ Every actor performs at the playhouse every evening.

(b) *The/ Every actor perform at the playhouse every evening.

While the observed P600 effect aligns with prior research findings (Tanner and Bulkes, 2015; Armstrong et al., 2018), in the present study, this effect was delayed. The latency difference is attributed to L2 proficiency in L2 processing research (Hahne et al., 2006; Rossi et al., 2006). For example, Rossi et al. (2006) examined the influence of proficiency on late second language processing by using an auditory ERP paradigm. They find that at high proficiency, late L2 learners exhibit the native-like ERP pattern, which contains an early anterior negativity (ELAN) and a subsequent P600 effect. By contrast, L2 learners of low proficiency level only display the delayed P600 effect. Our participants’ English proficiency was lower than that of participants in previous studies (Tanner and Bulkes, 2015; Armstrong et al., 2018), which explains the delayed P600 effect observed in our study. Some research on English processing of Chinese learners indicates that intermediate learners show a relatively slow reading speed with each word at approximately 500 ms (Wu, 2016; Wu et al., 2018). Similarly, the participants in our study might be slower in initiating the cognitive processes underlying the P600s. Another possible reason for the P600 latency difference may be the different presentation times. In our study, each word was presented for 500 ms, with a 350-ms interval between words. By contrast, Tanner and Bulkes (2015) and Armstrong et al. (2018) set the presentation time for each word to 300 ms and the blank screen for the interstimulus interval to 200 ms. The longer word presentation time in our study might delay the latency of the P600 effect.

### 4.2 The modulation of the determiner type on English subject–verb agreement processing

Determiners differ concerning D-linking and number specification, leading to different neurocognitive processing patterns: D-linking leads to differences in scalp distribution of P600 and number specification gives rise to distinctive P600 latency and ERP components. Next, we will discuss in detail the effects of D-linking and number specification on the processing of English subject–verb agreement violations with omission errors among Chinese L2 learners.

The difference in D-linking between the referential determiner and the quantificational determiner has been demonstrated to surface with topographically different positivities. Subject–verb agreement violations in the QDP conditions elicit laterally and frontally distributed P600 effects. The frontal positivities are proposed to be associated with integration complexity at the discourse level (Kaan and Swaab, 2003). Quantifiers (e.g., *each*) are D-linked and hence require interpretive links to members of a presupposed set that comprehenders have in mind. Integration of discourse-related knowledge gives rise to an increase in the frontal portion of the P600 effect. By contrast, agreement violations in the RDP conditions result in posteriorly distributed P600 effects, which are consistent with previous studies on subject–verb agreement processing (Friederici, 1995; Osterhout and Mobley, 1995; Nevins et al., 2007). The posterior P600 component is held to reflect sentence reanalysis (Friederici, 1995; Tanner et al., 2017) or difficulty with syntactic integration (Kaan et al., 2000). Non-D-linked referential determiners (e.g., *the*) are determined solely by syntactic constraints, with no reference to discourse representations, and as such, elicit the posteriorly distributed P600 effects.

ERP patterns vary as a function of the number specification of different determiners. While both the RDP and QDP conditions exhibit P600 effects, the onset latency of this effect is delayed in the un-quantificational RDP condition compared to the QDP condition. Moreover, the RDP condition also displayed an additional N400 component. The earlier P600 onset latency for the QDP condition may be the result of greater ease of retrieval, temporal advantage, and certainty concerning the causes of the agreement errors. First of all, in terms of greater ease of retrieval, quantifiers (e.g., *each*/*every*) possess a specified number feature of singular, whereas referential determiners (e.g., *the*) do not specify the number. In the QDP conditions (e.g., *each boy*), the additional quantificational feature or double number marking of DP by a singular quantifier renders a stronger sensitivity to subject–verb agreement violations. The onset latency of the P600 effects was earlier when the subject DP is marked as singular by a singular quantifier *each*/*every* relative to when it remains unmarked by the referential determiner *the*. Earlier P600 effects elicited by subject–verb agreement violations in the QDP condition are compatible with the cue-based retrieval model’s prediction that the double marking of the singular number by a singular quantifier will render a strong predictive cue to the upcoming singular verb. The current results share some characteristics with predictions made by Tanner and Bulkes (2015), who suggest that with the help of plural quantifiers, comprehenders can instantly start predicting the number feature of an incoming verb. These findings are in line with the accounts of the cue-based retrieval model. According to the model, the RDP and QDP should behave differently when retrieved due to differences in the markedness of the number feature. For example, in sentence 5(a), after the singular subject noun *house* is encountered, its number feature [+Singular] is stored in memory, and the retrieval cue [-Singular] provided by the verb disagrees with the subject feature [+Singular]. The increase in memory loads caused by retrieval failure elicits a P600 effect. In 5(b), the double number encoding of *every house* offers a more effective disambiguating cue to the number feature of subject QDP than the unmarked RDP *the house* does. It is suggested that the onset latency of the P600 effect is associated with the ease of detecting the anomalies, retrieving the specific features from working memory, and completing sentence reanalysis (Gouvea et al., 2010). In this case, it takes less time to recognize the upcoming verb and access the number feature of the DP from working memory in the overtly singularly quantified DP conditions than in the unmarked DP conditions. Therefore, both the RDP and QDP conditions exhibit a P600; however, its onset latency was later in the RDP condition relative to the QDP condition.

5 (a) *The house get repainted every summer.

(b) *Every house get repainted every summer.

As far as a temporal advantage is concerned, the presence of a singular quantifier in the QDP *every house* helps comprehenders anticipate a singular verb in advance, even before the noun is encountered since singular quantifiers only occur with singular verbs. By contrast, the number-ambiguous determiner *the* does not possess number specification, and it can be followed by either a singular noun or a plural noun. The temporal advantage provided by the quantifiers facilitates an earlier anticipation of a singular verb than the unmarked determiner *the*. Furthermore, the earlier P600 onset latency in the marked QDP conditions can also be attributed to L2 learners’ certainty with causes of agreement errors. Subject–verb agreement violations can only be the result of the omission of the 3rd person singular *-s* inflection on the verb since singular quantifiers specify a singular subject, e.g., every house *get/gets repainted…. By contrast, causes of agreement errors can be attributed to either the omission of *-s* on the verb, e.g., the house gets/*get repainted…, or the omission of the plural marker -*s* on the subject, e.g., the *house/houses get repainted…, because the number-ambiguous *the*, which lacks a grammatical feature for number, can occur with either a singular noun or a plural noun.

Additionally, number specification also results in different processing patterns observed in the RDP and QDP conditions. For the marked QDP conditions, the native-like P600 component is evoked in response to subject–verb agreement violations, which is in line with findings from previous studies in native speakers (Osterhout and Mobley, 1995; Tanner et al., 2013; Tanner and Bulkes, 2015). The P600 effect observed in QDP conditions indicates successful acquisition of this specific dependency and native-like grammatical processing, demonstrating that Chinese L2 learners can exhibit a native-like P600 effect in response to agreement violations, even though the rule is absent in their L1. This finding has pedagogical implications for subject–verb agreement acquisition. In the textbooks, we suggest that subject–verb agreement materials are used, which contain doubly number-marked DPs by a quantifier. In classroom instruction, teachers can provide more language input with explicitly number-marked DPs functioning as subjects.

For the RDP conditions, however, an additional N400 effect is observed for agreement violations. The N400 is claimed to be sensitive to novel word combinations or word sequence probabilities (Kutas and Hillyard, 1984). Osterhout et al. (2006) reported that an N400 effect is elicited in response to the subject–verb agreement violation *tu adorez in L2 English learners of French, which is due to syntactically ill and novel word combinations. Similarly, such unfamiliar and syntactically ill word combinations as subject–verb agreement *the boy like* in the RDP condition elicit an N400 component in our L2 Chinese learners of English. Another possibility might be that L2 learners acquire morphologically complex words by rote and memorize them as unanalyzed chunks (Myles et al., 1998). Specifically, Chinese L2 learners initially memorize that specific subjects come after specific forms of verbs, e.g., *the boy* is followed by *likes,* whereas *the boys* is followed by *like*; thus, they cannot decompose *likes* into root *like* + morpheme *–s* or induce such a morphosyntactic rule. At this stage, L2 learners associate meanings with the undecomposed word and exhibit an N400 effect.

## 5 Conclusion

Our study investigated the modulation of different determiners on the processing of L2 subject–verb agreement with omission errors. Two major findings are as follows: On the one hand, subject–verb agreement violations elicit a P600 in Chinese learners without immersive learning experiences, which is in line with the previous research (Tanner and Bulkes, 2015; Armstrong et al., 2018). Based on the cue-based retrieval accounts, we provide a new interpretation of the P600 effect: P600 is elicited due to retrieval failure during the retrieval processes. On the other hand, features of different determiners influence the neurocognitive processing of subject–verb agreement by Chinese learners of English, as is shown in greater variation in the latency or scalp distribution of ERP effects. These findings have pedagogical implications for subject–verb agreement acquisition, as well as for L2 acquisition and sentence processing theories. Different grammatical features vary in degrees of acquisition difficulty; even the same grammatical features (such as subject–verb agreement) have different acquisition and processing difficulties in various sentence environments. Therefore, formulations of adequate L2 acquisition and processing theory should cover different grammatical features and different distributions of the same grammatical features. The present study highlights two suggestions for future direction. First, more ERP studies are needed to identify whether different determiners influence the processing of subject–verb agreement with number attraction effects. Second, replication studies are encouraged, using neuroimaging methods such as fMRI and fNIRS to examine distributional variations in the hemodynamic response to increased discourse processing as a function of different determiners.

## Data availability statement

The original contributions presented in the study are included in the article/supplementary material, further inquiries can be directed to the corresponding author.

## Ethics statement

The studies involving humans were approved by the ad hoc human ethics review committee chaired by the Dean of the School of Foreign Languages in Jiangsu University of Science and Technology. The studies were conducted in accordance with the local legislation and institutional requirements. The participants provided their written informed consent to participate in this study.

## Author contributions

MW: Writing – review & editing, Writing – original draft, Visualization, Validation, Resources, Methodology, Investigation, Data curation, Conceptualization. ML: Writing – review & editing, Writing – original draft, Visualization, Validation, Software, Resources, Project administration, Methodology, Investigation, Data curation, Conceptualization. DW: Writing – review & editing, Writing – original draft, Methodology, Investigation, Conceptualization.
